# An Application of Data Mining Techniques to Explore Congressional Lobbying Records for Patterns in Pediatric Special Interest Expenditures Prior to the Affordable Care Act

**DOI:** 10.3389/fdata.2018.00003

**Published:** 2018-08-20

**Authors:** Elizabeth Harrison, Caitlin Dreisbach, Nada Basit, Jessica Keim-Malpass

**Affiliations:** ^1^Data Science Institute, University of Virginia, Charlottesville, VA, United States; ^2^School of Medicine, University of Virginia, Charlottesville, VA, United States; ^3^School of Nursing, University of Virginia, Charlottesville, VA, United States; ^4^Department of Computer Science, University of Virginia, Charlottesville, VA, United States

**Keywords:** lobbying, data mining, data science, Affordable Care Act, pediatrics

## 1. Background

Since 2006, 1.7 billion dollars has been spent lobbying Congress and federal agencies in the area of health (Steinbrook, 2009). Despite this, there has been a dearth of empirical studies focusing on the relationship between financial lobby influence and associated health legislation (Steinbrook, 2009). Most of the existing empirical examples assessing the impact of lobbying on legislation focus on the relationship between either lobbying and tobacco or pharmaceutical and medical device regulations (Steinbrook, 2009; Jorgensen, 2013; Savell et al., 2014). Associated concerns over transparency and public access to lobbying financial disclosures have only recently begun to be addressed (Steinbrook, 2009).

In the United States, there has been a long history of advocacy on the behalf of children (Denne and Hay, 2013). The year 1921 marked the first federal legislative effort (the Sheppard-Towner Act) to designate federal funds for prenatal programs targeting poor women and children (Denne and Hay, 2013). Since then, federal programs focused on child health have increased in scope and quantity, including numerous federal programs that expanded with the 2010 passage of the Patient Protection and Affordable Care Act (ACA) (Farrell et al., 2011; Keim-Malpass et al., 2013, 2015). This period, in particular, gave an opportunity for researchers and clinicians to advocate for their pediatric specific interests (Steinbrook, 2009).

The use of data science applications provides a scientific framework to describe the financial impact of legislation and lobbying for pediatric issues. Emerging trends in the use of data science methods within a social science domain have focused on social network analyses, surveying sentiment through text, and understanding patterns in publicly available datasets (Bellazzi et al., 2011; Asch et al., 2015). A recent study in the European Union on quantitative textual analysis highlighted the importance of rich, public data sources that have the potential to highlight incongruities between lobbying positions and legislative success (Bunea and Ibenskas, 2015). In the United States, the Lobbying Disclosure Act (LDA), signed into law by President Bill Clinton, requires the Secretary of the Senate and the Clerk of the House of Representatives to make publicly available all registrations and reports related to lobbying activity^1^. The intended purpose of this act was to increase the accountability of federal lobbying practice. However, the relatively unexplored nature of the LDA database suggests the need for advanced methods to provide clarity to the power of lobbying.

Analyses of congressional lobbying activities focused on child health prior to the ACA offer a unique perspective on the various factors that contribute to child health advocacy and the eventual passage of relevant legislation. This data report illustrates several data parsing principles that can be used to mine lobbying records and can serve as the basis for future inquiry and analysis.

## 2. Data and methodology

Data science techniques such as parsing, cleaning, and transforming raw data to identify patterns and trends can be used to focus on important lobbying issues published through the LDA. Coding languages –s in this case Python – can be critical tools to extract meaningful and actionable items within troves of data (Wiedeman, 2017). As a dynamically-typed and general-use programming language, Python provides a new technical toolbox for political researchers, clinicians, and data scientists. By leveraging data science for the investigation of lobbying for pediatric research and clinical trials, this data report aims to illustrate how one might investigate deeper trends regarding the inclusion of pediatric health concerns in the political environment.

### 2.1. Data source

This data report focuses on data pertaining to lobbying records submitted in the fourth quarter of 2009, a period of intense healthcare lobbying prior to the passage of the Affordable Care Act. Currently, the public LDA database can be accessed online. The website provides both a search engine for specific database queries and downloadable copies of the raw XML data from 1999 to the current year^2^. The data from each quarter comprises about 25 separate congressional lobbying XML files with approximately 1,000 lines of code each. Thus, the final dataset used for the analysis in this report includes over 25,000 lines of XML code.

### 2.2. ElementTree and XML overview

**Figure 1:**
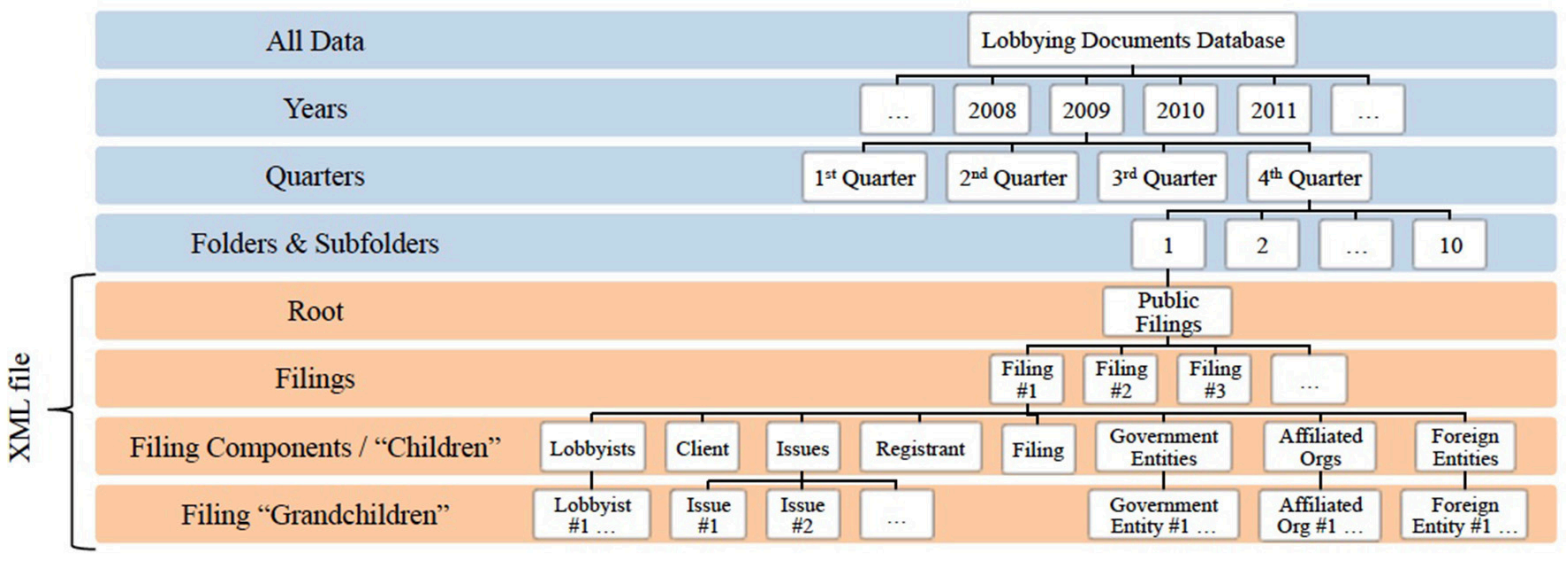
Data organization tree.

The authors' extraction method uses a popular web-parsing package in Python called ElementTree. The ElementTree (ET) module utilizes a simple and efficient Application Programming Interface (API) for parsing, sorting, and organizing XML data^3^. Extensible Markup Language (XML) was designed for widespread use across the internet, and is readable by both humans and machines^4^. ET runs through the naturally hierarchical format of XML files to parse out root and child attributes, which can then be used to sort the information into other data structures. For reference, the organizational structure of the data is outlined in Figure 1, including the component extracted for this data report.

### 2.3. Data preprocessing

**Figure 2:**
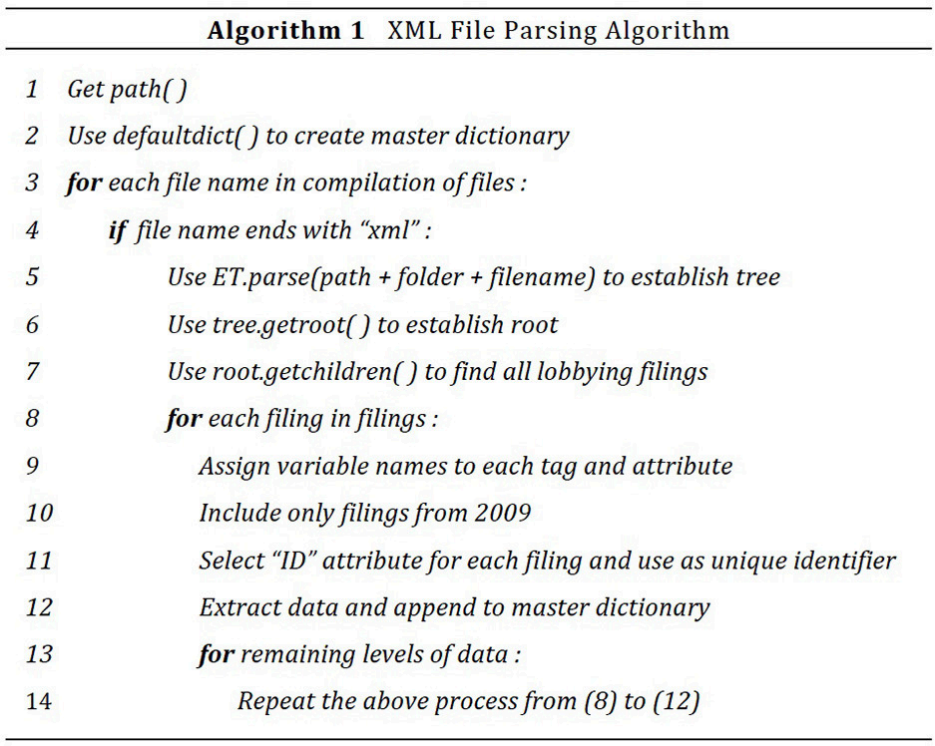
XML parsing algorithm pseudocode.

To begin, all necessary packages are loaded, including ElementTree for parsing, Pandas for the creation of multiple data frames, and defaultdict to organize the central dictionary for the soon-to-be parsed data. Next, the LDA site is accessed to download the folder with lobbying data from the fourth quarter of 2009. An os.listdir function is applied to extract all individual files from this folder. Finally, an algorithm is used to parse through the XML files and convert the congressional lobbying data into a more usable form. See Figure 2 for this algorithm in pseudo-code.

An initial data organization issue related to lobbying records is that these records are grouped by time of filing, not by the time that the contribution took place. For example, the lobbying records from the fourth quarter of 2009 include multiple records that originated from years between 2002 and 2008 but were filed after the fact. To account for this, a special statement is included in the algorithm to ensure that the extracted data includes events from the year 2009 alone.

After extracting the data, but prior to performing any analysis, a master dictionary is used to create eight individual data frames, one for each of several keys in the master dictionary: “Filing,” “Issue,” “Entity,” “Org,” “Lobbyist,” “Registrant,” “Client,” and “Government Entity.” Subsequently, another data frame is created by merging “Filing,” which includes amounts of money reported per filing, and “Issue,” which includes umbrella codes and more specific issues of interest. Finally, a Pandas series is formed from the new data frame by grouping amounts by code.

### 2.4. Queries and searching

To set up the code structure for more specific queries, a search function is developed to identify output based on user input. This function creates an empty list and appends each dictionary item that contains the search term in its “Specific Issues” section, which is a subcategory of each individual “Issue” section. Because the search is based on user input, any term can be searched within the list of dictionaries. The final appended list can then be easily converted into a data frame.

Consideration of a wide variety of related terms is essential in the creation of an effective initial query. For the purposes of this data report, the term “pediatric” is chosen because it is both sensitive and specific for medical data in the under 18-year-old population. Using this single term, 35 results are pulled from the entire fourth quarter data frame. Then, key pieces of action and legislation are identified, such as the Improving Access to Clinical Trials Act of 2009, programs to expand medical device testing in children, and budget appropriations for generic pediatric drug trials. Finally, the search results are compared to the previously saved data regarding codes and lobbying dollar amounts spent per issue.

## 3. Analysis

A sorted amount-by-code Pandas series shows that the largest sum of lobbying dollars spent and filed in the fourth quarter of 2009 has been filed under “Taxation/Internal Revenue Code,” with an associated value of $527,147,333. Next is “Health Issues,” with $436,651,121.

Analysis of the pediatric search results shows that these lobbying disclosures have been filed under a multitude of umbrella codes, including “Health Issues,” “Budget Appropriations,” “Science/Technology,” “Education,” “Disaster Planning & Emergencies,” and “Medicare/Medicaid.” Although this data can be merged with the “Filing”/“Issue” data frame, it remains impossible to make meaningful deductions about amounts spent on pediatric lobbying activities specifically because multiple issues are grouped together with the pediatric-related items, such as one filing that cites the following specific issues in one group:

“Support legislation to amend title XVIII of the Social Security Act to provide for coverage of federally recommended vaccines under Medicare, Part B. Support legislation to amend title XIX of the Social Security Act to include all public clinics for the distribution of pediatric vaccines under the Medicaid program. Support legislation to amend the Social Security Act, the Federal Food, Drug and Cosmetic Act, and the Public Health Service Act to ensure a sufficient supply of vaccines and for other purposes. Lobbied to ensure that the Internet would not be used to import counterfeit and defective medical products. Support a rigorous and workable Affordable Biologics for Consumers Act. Support enactment and follow-on Biologics legislation with amendments that strengthen FDA pathway and provide data exclusivity. General matters related to vaccine industry stability, pandemic preparedness, avian influenza”^4^.

Another unexpected but important finding of this data parsing method relates to the actual dollar amounts that can be extracted from the XML files. First, and most importantly, the XML files do not differentiate lobbying expenditures from income; these amounts are indistinguishable and are not linked to any identifiers that would allow a researcher to parse them out separately. Second, although the amounts appear to be specific to the single dollar on the results from the queries, the forms used to file lobbying reports allow a box to be checked for all lobbying expenses under $5,000, without any space to indicate the actual amount. Finally, in regards to income from clients, the forms used to gather lobbying data allow amounts over $5,000 to be rounded to the nearest $10,000:

“Provide a good faith estimate, rounded to the nearest $10,000, of all lobbying related income from the client (including all payments to the registrant by any other entity for lobbying activities on behalf of the client)”^5^.

Due to the clear inefficiency of the original reporting forms, this report identifies concerns with the process of lobby reporting at its source. Although the current public LDA database is searchable and the raw XML data is available to download, the system continues to lack transparency.

## 4. Future research

Pediatric research is a topic for which critical analysis of lobbying records may be clinically meaningful, particularly with regards to records originating at the time of the ACA's passage. Further, supporting the development of major healthcare bills that influence patients across the age continuum is essential to countering current governmental efforts to decrease healthcare appropriations.

Future research could leverage other data science techniques to assess the value of the underlying text in lobbying records. Potentially helpful methods include natural language processing (NLP), topic modeling, and generalized text analysis algorithms. Refining methods to the text level may illuminate greater detail about inconsistencies in the way lobbying records are written. However, while a greater understanding of lobbying language might be beneficial, this kind of analysis would largely ignore the immense financial representation that lobbying groups have in the United States Congress.

More importantly, deeper analytical processes – including greater statistical manipulation of the currency values that are associated with each lobbying record – could be assessed. As mentioned in our results, the limitation in greater statistical analyses is the innate inability to identify the exact amount of money spent on any particular lobbying activity. Only when the forms and procedures used to collect lobbying data are adjusted will data mining techniques be helpful in shedding light on federal lobbying practices, both in and out of the healthcare policy realm.

## Data availability statement

The datasets analyzed for this report can be found on the United States Senate website: https://www.senate.gov/legislative/Public_Disclosure/database_download.htm. All pertinent code is accessible via Github: https://github.com/eih2nn/UnitedStatesLobbyingDisclosureData.

## Author contributions

EH and CD contributed to conception, design, analysis, and interpretation. EH parsed the data, led all coding, and developed the extraction algorithm. CD wrote the first draft of the report. JK-M and NB contributed to concept development and review. All authors contributed to manuscript revision and read and approved the submitted version.

### Conflict of interest statement

The authors declare that the research was conducted in the absence of any commercial or financial relationships that could be construed as a potential conflict of interest.

## Footnotes

^1^104th Congress. Lobbying Disclosure Act of 1995. Available online at: https://lobbyingdisclosure.house.gov/lda.html.

^2^US Senate Office of Public Records US. Lobbying Disclosure Act (LDA) Downloadable Lobbying Databases (2017). Available online at: https://www.senate.gov/legislative/Public_Disclosure/database_download.htm.

^3^20.4. XML Processing Modules - Python 3.6.4 documentation (2018). Available online at: https://docs.python.org/3/library/xml.html.

^4^20.5. The ElementTree XML API (2018). Available online at: https://docs.python.org/3/library/xml.etree.elementtree.html.

^5^Lobbying Registration (LD-1DS) Sample Form (2017). Available from: https://lobbyingdisclosure.house.gov/help/default.htm?turl=WordDocuments%2Flobbyingregistrationld1dssampleform.htm.
